# Nurse Aide Turnover in Long-Term and Acute Care Settings: Workplace Dynamics, Incontinence Care, and Job Demands

**DOI:** 10.1093/geroni/igaf122.3032

**Published:** 2025-12-31

**Authors:** Vivian Miller, Lauren Maziarz, Eric Cooke, Julia Bell, Jennifer Wagner, Melissa Burek

**Affiliations:** Bowling Green State University, Bowling Green, Ohio, United States; Bowling Green State University, Bowling Green, Ohio, United States; Bowling Green State University, Bowling Green, Ohio, United States; Northeastern University, Boston, Massachusetts, United States; Bowling Green State University, Bowling Green, Ohio, United States; Bowling Green State University, Bowling Green, Ohio, United States

## Abstract

Turnover rates among Nurse Aides (NAs) within long-term and acute care settings are incredibly high; annual turnover rates within these settings exceed 100%. Nurse Aide (NA) turnover at this rate jeopardizes resident care and places increase burden on remaining staff within these settings. Much research has focused on working conditions and staffing ratios as impacting NA turnover but has yet to examine specific challenges including incontinence care and workplace dynamics. Guided by the Triple Aim, Quadruple Aim, and Quintuple Aim frameworks and the socio-ecological model, this study utilizes a cross-sectional survey to analyze the relationship between perceived workplace challenges, time spent on incontinence care, and turnover intentions. Findings from a sample of 307 NAs (N = 307) reveal that time spent on incontinence care significantly predicts perceptions of work being challenging but is not directly associated with turnover intentions. Rather, factors such as physical demands, workplace culture, and poor management support were more predictive of turnover. These results underscore the complexity of NA turnover within long-term and acute care settings. Workplace culture, physical demands, management support, and incontinence care are found to be challenging and may impact NAs intention to leave their job. Efforts to reduce turnover among NAs should focus on addressing broad workplace challenges, including incontinence care practices, such as NA training and assistive equipment. Future research may examine incontinence care and workplace considerations over time. Policies may consider initiatives to reduce disparities in NA turnover and improve overall quality of care within and across long-term care settings.

